# Production of soluble eukaryotic recombinant proteins in *E. coli* is favoured in early log-phase cultures induced at low temperature

**DOI:** 10.1186/2193-1801-2-89

**Published:** 2013-03-08

**Authors:** Teresa San-Miguel, Pedro Pérez-Bermúdez, Isabel Gavidia

**Affiliations:** Departamento de Biología Vegetal, Facultad de Farmacia, Universidad de Valencia, Av. VA Estellés s/n, Burjasot, 46100 Spain

**Keywords:** Early log phase, Functional proteins, Low temperature, Soluble recombinant proteins

## Abstract

**Background:**

Producing recombinant plant proteins expressed in *Escherichia coli* produce in high yields and in a soluble and functional form can be difficult. Under overexpression conditions, proteins frequently accumulate as insoluble aggregates (inclusion bodies) within the producing bacteria. We evaluated how the initial culture density, temperature and duration of the expression stage affect the production of some eukaryotic enzymes in *E. coli*.

**Findings:**

A high yield of active soluble proteins was obtained by combining early-log phase cultures and low temperatures for protein induction. When IPTG was added at OD_600_ = 0.1 and cultures were maintained at 4°C for 48-72 h, the soluble protein yield was 3 fold higher than that obtained in the mid-log phase (OD_600_ = 0.6). Besides, the target protein expression increased and the endogenous bacterial proteins reduced, thus making the protein purification process easier and more efficient.

**Conclusions:**

The protocol can be widely applied to proteins with a heterologous expression which was limited by loss of activity at high temperatures or by low soluble recombinant protein yield.

## Findings

### Background

One important limitation for the production of recombinant proteins in *Escherichia coli* is obtaining large amounts of soluble and functional proteins. Under overexpression conditions, proteins frequently accumulate as insoluble aggregates (inclusion bodies) within producing bacteria. The production of soluble proteins under native conditions is essential for functional and structural analyses. Therefore, many studies have focused on optimising processes in protein expression (see Peti and Page 2007). One possibility is the solubilisation of proteins from inclusion bodies, but it usually requires denaturing conditions, and the subsequent renaturing step can prove difficult. Several experimental approaches have been developed to prevent these aggregates from forming, which include the use of cold inducible expression systems (Qing et al. 2004, Thuy Le and Schumann 2007), to increase the intracellular concentration of molecular chaperones (Mogk et al. 2002), to reduce the IPTG concentration for induction (Winograd et al. 1993), to induce the expression in a late log phase culture (Galloway et al. 2003), and to lower the growth temperature of induced cultures (Schein and Noteborn 1988, Vera et al. 2007).

 In previous works, we reported the isolation and biochemical characterisation of genes *P5βR* (Gavidia et al. 2007) and *P5βR2* (Pérez-Bermúdez et al. 2010), which encode progesterone 5β-reductase (P5βR). These enzymes catalyse the 5β-reduction of progesterone to 5β-pregnan-3,20-dione as the first committed step in the biosynthetic pathway leading to cardiac glycosides in *Digitalis* species. In order to increase active enzyme yield, this study evaluated the effects of different temperatures and induction periods, and of varied initial culture densities, on the recombinant P5βR2 expression levels.

### Methods

The full-length open-reading frame of *P5βR2* cDNA was amplified by PCR to include the *Pst*I restriction site at the 3^′^ end by using Pfu DNA polymerase. The product was subcloned into the *Xmn*I*-Pst*I sites of the pMAL-c2 vector (New England Biolabs) and was expressed as a fusion protein with the maltose-binding-protein (MBP) at the N-terminus. The constructed vector was transformed into *E. coli* BL21(DE3)pLysS. An overnight 3 ml culture from a single colony was grown at 37°C in LB medium containing 100 mg/l of ampicillin, and was then transferred to 25 ml LB supplemented with glucose (2 g/l) and ampicillin. After incubation at 37°C, the culture volume was increased to 150 ml. Gene expression was induced by the addition of 0.3 mM IPTG to the cultures with different cell densities. Expression cultures were grown in Erlenmeyer flasks (250 ml) with vented caps (PTFE membrane, 0.22 μm pore size). Protein production was evaluated in cultures which were induced when OD_600_ was 0.1 (early log phase), 0.6 (mid log phase) and 1 (late log phase). Cultures were placed at 37, 25, 15 or 4°C and were shaken at 200 rpm for different incubation periods (2-72 h). Before the protein expression induction, cultures were cooled until they reached their respective growth temperatures.

After induction, cells were harvested by centrifugation at 4,000 rpm for 20 min at 4°C and were frozen at -20°C. Cells were disrupted by sonication in buffer A (20 mM Tris–HCl pH 7.4, 200 mM NaCl, 1 mM EDTA, 1 mM DTT and 0.2 mM PMSF), and soluble protein extracts were obtained after centrifugation at 12,000 g for 10 min. The MBP-tagged P5βR2 protein was then purified to apparent homogeneity in buffer B (buffer A with 10 mM maltose) by amylose resin column chromatography (New England Biolabs), following the supplier’s protocol. The electrophoretic separation of proteins was performed on 12% polyacrylamide gels according to Laemmli (1970). Proteins were quantified by the method described by Bradford (1976). Experiments were performed in triplicate. The study was approved by the institutional Ethics Commision in Experimental Research of the University of Valencia (Spain).

### Results and discussion

Progesterone 5β-reductase belongs to the short-chain dehydrogenase/reductase (SDR) superfamily, and has been considered a key enzyme in cardenolide biosynthesis since it is the first stereospecific enzyme of the pathway leading to 5β-configured intermediates (Roca-Pérez et al. 2004, Gavidia et al. 2007, Pérez-Bermúdez et al. 2010).

Our results demonstrated that obtaining this steroid reductase in a functional form absolutely depends on the temperature at which induction is performed. The induced cultures grown at 37°C (for 2 h or 3 h) or at 25°C (for 4 h or 6 h) did not produce active protein at any density. Thus only the induced cultures grown at 15°C, or at lower temperatures, produced the active MBP-P5βR2 fusion protein (for details about enzyme activity, see Pérez-Bermúdez et al. 2010). Moreover, using the cultures of OD_600_ = 1 for protein induction did not improve the results obtained with the cultures of OD_600_ = 0.6 irrespectively of whether the expression was carried out at 15°C or 4°C, the yields of soluble protein obtained in both trials were almost identical.

**Figure 1:**
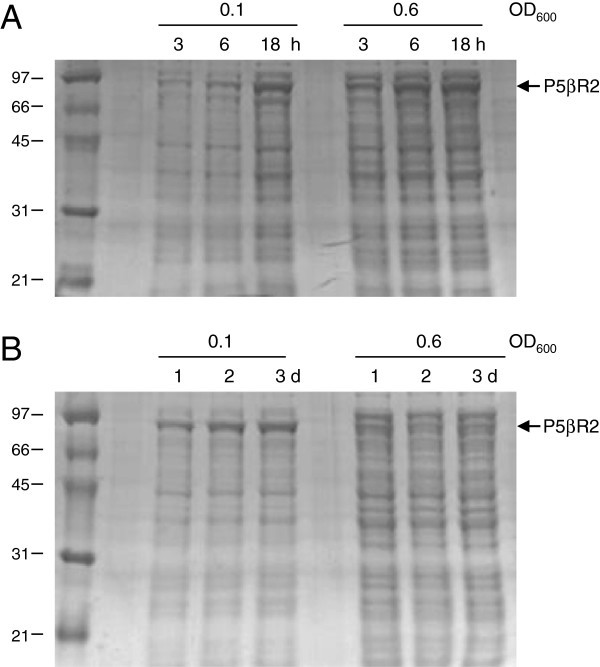
**Expression of soluble recombinant P5βR2 at low temperatures.** The *E. coli* strain BL21 (DE3) pLysS carrying plasmid pMal-P5βR2 was grown in LB medium at 37°C. The heterologous protein expression was induced in the early- (0.1) or mid-log- (0.6) phase culture with 0.3 mM IPTG for the indicated time period at 15°C (**A**) or at 4°C (**B**). Cells were lysed by sonication and an equal aliquot of soluble protein was resolved by SDS-PAGE. Figure 1B has been adapted from Pérez-Bermúdez et al. (2010).

**Figure 2:**
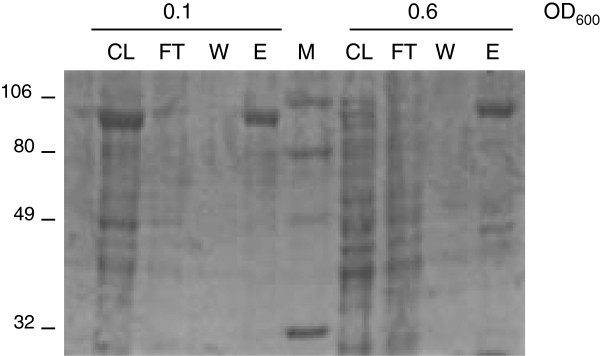
**Purification of P5βR2 by amylose resin column chromatography.** Soluble protein isolated from early and mid log phase cells which were induced at 4°C. CL, cell lysate; FT, unbound protein in the flow through; W, wash; E, 3.5 μg of eluted protein.

Within the 15°C and 4°C temperature range, the highest soluble protein yield was obtained at 18 h and 72 h, respectively. When protein induction was carried out at 15°C, the production rate was similar at both cell densities, the early or mid log phase, after 18 h of incubation (Figure 1A). At this temperature, the cultures induced at the early-log phase presented a slightly low endogenous protein production (Table 1). However, when the protein expression commenced in the bacteria grown at 4°C, the most significant soluble P5βR2 production was obtained in those cultures induced in the early log phase. Thus, when IPTG was added at OD_600_ = 0.1 and cultures were maintained at 4°C for 48-72 h, the amount of soluble protein was 2-3-fold higher (Table 1) than that obtained when IPTG was added in the mid-log phase (OD_600_ = 0.6) (Figures 1B and 2). Under these conditions, the purified P5βR2 recovered from 150 ml of culture was 3-4 mg, and the expression of the endogenous bacterial proteins lowered 30% (Table 1). Thus, the protein purification process proved more amenable and efficient. P5βR2 synthesis was maintained for 72 h after induction (Figure 1B), indicating that cells under those conditions retained the protein-synthesising capacity for more than 3 days.

**Table 1:** **Quantitative overview of recombinant P5βR2 purification in the early- (0.1) or mid-log- (0.6) phase culture**

Culture density	4°C/72 h			15°C/18 h		
	P5βR2 (mg)	Total protein in cell lysate (mg)	Yield (%)	P5βR2 (mg)	Total protein in cell lysate (mg)	Yield (%)
OD_600_ = 0.1	3.6 ± 0.2	12.1 ± 1.3	30	3.5 ± 0.3	21.4 ± 1.9	16
OD_600_ = 0.6	1.4 ± 0.2	20.8 ± 3.4	8	3.8 ± 0.4	23.0 ± 2.8	17

*Escherichia coli* cultures were incubated at low temperatures (4°C or 15°C) for the indicated time period. Data are the mean values (±SD) from three separate 150 ml expression cultures.

It is known that the overexpression of the proteins in *E. coli* at low temperatures usually improves both protein solubility and activity (see Sahdev et al. 2008). Accordingly, it has been reported that preferred induction temperatures would fall in the 16°C - 23°C range (Niiranen et al. 2007, Vera et al. 2007, Peti and Page 2007), but also at around 10°C (Vasina and Baneyx 1997, Pacheco et al. 2012). Yet, as far as we know, protein expression in early log phase cultures (OD_600_ = 0.1) has not been reported. Low growth rates are usually associated with a low protein synthesis rate, which is consistent with our observations for the production of endogenous bacterial proteins, but not with recombinant protein yield. It is noteworthy that, the data presented herein demonstrate that the high soluble protein yield from the early log-phase cultures, grown at temperatures as low as 4°C, does not correlate with a high cell number (Table 1). Although 4°C is a low temperature, the bacterium grows. Actually, an *E. coli* culture induced to generate the recombinant P5βR2 protein (OD_600_ = 0.1) produced a biomass of ca. 45-50 mg fresh wt/150 ml cultivation broth in 72 h at 4°C. This growth cannot be associated with the recombinant P5βR2 expression since *E. coli* cultures showed a similar growth rate when bacteria were transformed with the empty vector. In line with this, there have been reports that the expression of some heterologous proteins (chaperonins) determine *E. coli* growth at low temperatures; thus, transgenic strains grew at temperatures below 4°C and the theoretically minimum temperature for growth would be as low as -13.7°C (Ferrer et al. 2003).

We also observed that slow-growing cultures were more permissive for expressing heterologous proteins than rapid-growing ones in which protein translation is maximal. In our trials, this behaviour was not exclusive of the pMAL system and the BL21(DE3)pLysS strain, since we obtained similar production rates using the pQE (Qiagen) and pET (Novagen) systems with different *E. coli* strains, such as M15[pREP4], SG13009[pREP4] and BL21(DE3).

**Figure 3:**
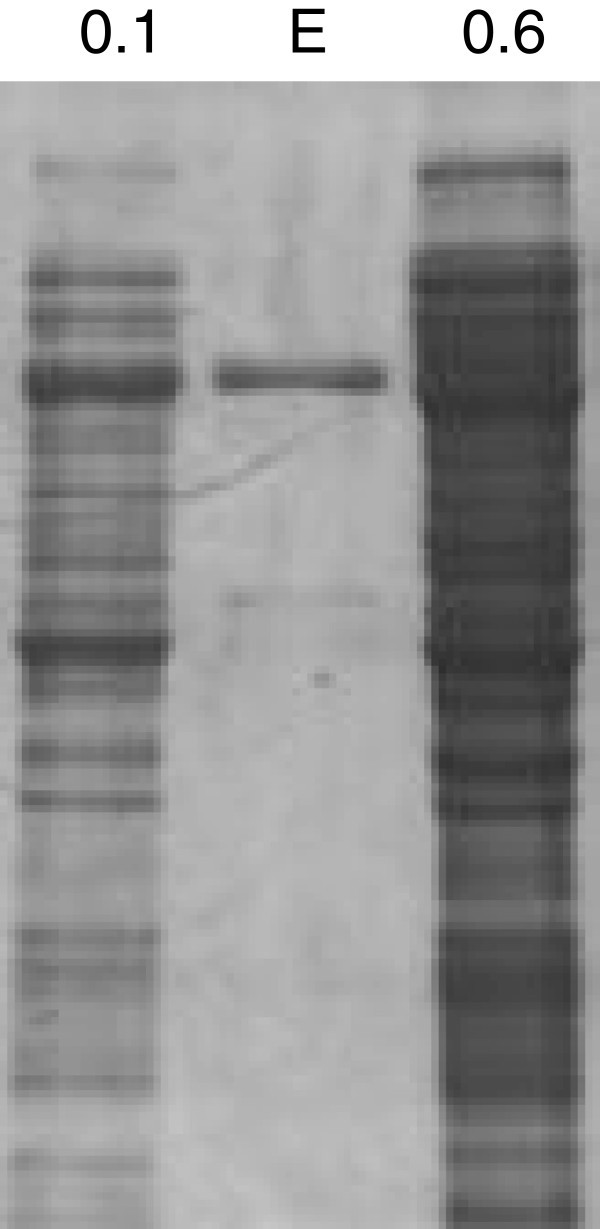
**Expression of*Echinostoma caproni*HSP70 using the pQE30 expression vector and*E. coli*M15[pREP4] strain.** The heterologous protein expression was induced in the early- (0.1) or mid-log- (0.6) phase culture with 0.3 mM IPTG at 4°C. Cells were lysed by sonication and an equal aliquot of soluble protein was resolved by SDS-PAGE. E, 3.5 μg of eluted protein.

Finally, our protocols have also been incorporated into protocols for the expression of proteins from other organisms, such as HSP70 from the parasite *Echinostoma caproni. E. caproni* HSP70 is in part soluble and another part is insoluble. Furthermore, its expression in *E. coli* required the use of 8 M urea to improve the solubility of the heterologous protein (Higón et al. 2008). In the present trial, the heterologous expression of *E. caproni* HSP70 was induced in early- (0.1) or mid-log-phase (0.6) cultures at 4°C. The obtained results demonstrate the successful application of the protocol and that protein expression is favoured in early log-phase cultures induced at low temperatures (Figure 3) as compared with typically recommended culture conditions (OD_600_ = 0.6). Therefore, this protocol may be widely applied to proteins with a heterologous expression which is limited by loss of activity at high temperatures or by low soluble recombinant protein yield.
